# Gender, Stress, and Well-Being in Adulthood

**DOI:** 10.3390/jcm12010110

**Published:** 2022-12-23

**Authors:** J. Iván Pérez, M. Pilar Matud

**Affiliations:** Department of Clinical Psychology, Psychobiology, and Methodology, Universidad de La Laguna, 38200 San Cristobal de La Laguna, Spain

**Keywords:** gender, stress, eudaimonic-well-being, life satisfaction, life events, daily hassles, coping styles, masculine/instrumental trait, feminine/expressive trait, adulthood

## Abstract

Exposure to stressors may be one of the most critical components of health and well-being. Although research on stress and health abounds, most studies have focused on establishing that stress is harmful to physical and mental health whereas less attention has been paid to analysing the effects of stress on well-being. The main purpose of this study is to investigate the relevance of gender in the association of stress with well-being in adulthood. A cross-sectional study was conducted with 1578 women and 1507 men from the Spanish general population aged between 30 and 59. All participants were assessed by eight self-reports measuring chronic stress, life events, daily hassles, eudaimonic well-being, life satisfaction, masculine/instrumental and feminine/expressive traits, coping styles, and social support. Although stress does not affect women’s eudaimonic well-being, a greater number of life events and more daily hassles rendered lower life satisfaction in women. Men’s greater chronic stress was associated with lower eudaimonic well-being and life satisfaction; moreover, a greater number of life events was associated with men’s lower life satisfaction. We conclude that gender is relevant in the stress and well-being of adults as well as in the association between stress and well-being.

## 1. Introduction

Stress has been considered the “Health Epidemic of the 21st Century”, and it is recognized that its effect on physical and emotional health can be devastating [1]. Stress is directly or indirectly linked to seven of the leading causes of death in developed nations and contributes greatly to the burden of suffering in such nations while being linked to a wide range of morbidity [2]. Despite the frequent use of the word “stress” both at a popular and scientific level, there is no universally agreed scientific definition [2,3,4]; it is a term that has been used too broadly and sometimes in misleading ways [5]. Stress research generally differentiates between stressors, which are the events and conditions causing subsequent reactions; perceived stress, which reflects the perception and appraisal of stressors; and strains, which refer to the physiological, psychological, and behavioural outcomes of perceived stress [4].

It Is recognized that exposure to stressors in daily life or throughout the life course may be one of the most critical components of health and well-being [6]. Several types of naturalistic stressor assessments have predominated in the research literature [6,7]: (1) life events, which refer to exposure to unusual or demanding events that have the capacity to change the patterns of life, are harmful or threatening, or that interrupt important goals [8]. From this perspective, the basis of experienced stress is an event that generates a need for physical, social, or psychological readjustment [6]; (2) chronic stressors, which refer to the presence of long-term or recurrent difficulties and strains that affect one or many areas of life [6]; (3) minor daily hassles, which are relatively minor and usually less emotionally arousing events [6].

Although there is evidence that stressors have a major influence on mood, behaviour, sense of well-being, and health, the association between psychosocial stressors and disease is affected by the number, nature, and persistence of said stressors as well as by the individual’s biological vulnerability, psychosocial resources and learned coping patterns [9]. Coping refers to the ways in which people actually react to and deal with stressors in their daily lives, including constructive responses, such as effort, problem-solving, help-seeking, distraction, or accommodation, as well as maladaptive responses, such as helplessness, opposition, rumination, escape or social isolation [10]. Lazarus and Folkman [11] considered coping as the thoughts and actions that people employ in stressful situations and defined two major coping types: emotion-focused coping, which relates to the regulation of emotions generated by the appraisal process of stressors; and problem-focused coping, which refers to the management of the problem and taking steps to remove or evade the stressor.

Coping is one of the variables that has been considered most relevant in research on the stress–health process. While a portion of the response to stress is influenced by aspects of biological heritage and the characteristics of the physical and social environment over which the person has little or no control, the coping responses are, at least potentially, under the person’s control with coping being one of the few variables in the stress process that lends itself to intervention [12]. Another important variable in the stress–health process is social support, which has also been considered a way of coping [13]. The tripartite model of coping [13] includes problem-focused coping, as mentioned, which refers to the direct efforts to solve the problem, emotion-focused coping, focusing on managing emotions, and relationship-focused coping, which focuses on maintaining close relationships during periods of stress. Although there is diversity in social support concepts and measures [14,15], perceived social support has emerged as a prominent concept [14]. Perceived support was defined as support that is available if in need [15] and characterizes social support “as the cognitive appraisal of being reliably connected to others” ([14], p. 416). Perceived social support is one of the most confirmed psychosocial factors influencing health outcomes [16,17] and has been consistently found to have negative relationships with distress and other measures of stress and strain [14,17,18,19,20].

Although research on stress and health abounds, most studies have focused on establishing that stress is harmful to physical and mental health [21], with less research focused on studying the effects of stress on well-being. Well-being refers to “optimal psychological functioning and experience” ([22], p. 142) and is generally considered to be multidimensional. Recent formulations of subjective well-being have proposed a tripartite taxonomy [23,24]: (1) affective or hedonic well-being, generally defined as the experience of positive affect along the absence of negative feelings and distress; (2) eudaimonic well-being, which is concerned with a sense of meaning and purpose in life, positive relations with others and with the realization of human potential [25]; (3) evaluative well-being being, or life satisfaction, that refers to “people’s explicit and conscious evaluations of their lives, often based on factors that the individual deems relevant” ([26], p. 49). Research has shown that subjective well-being and health are associated [23,24,26] although the three aspects (hedonic well-being, eudaimonic well-being, and life satisfaction) represent different facets of life experience and have different linked factors [25]. Some studies have documented the protective influence of eudaimonic well-being in reducing disease risks and promoting longevity [25,27,28,29,30]. Furthermore, hedonic well-being and life satisfaction can influence health [31], and a recently published study also found that life satisfaction was associated with reduced mortality among older adults [30].

Among the factors that seem to contribute to subjective well-being is stress exposure [24], although most research has focused only on the study of major life events. Research has revealed that major life events can have long- and short-term effects on subjective well-being, although the effects depend on the type of well-being assessed and the type of event [31,32]; for most events, the effects are stronger and more consistent on the cognitive component of well-being than on the affective component [31]. Research on the association between life events and life satisfaction has shown that major life events are associated with noticeable changes in life satisfaction and, for some events such as divorce and disability, changes are long-lasting [33,34,35].

Noticeable variations have been identified in the correlates of well-being across the life span [24], and, from a developmental perspective, life events are considered as specific transitions that mark the beginning or the end of a particular status [31]. Therefore, it is important that the study of the associations between stress and well-being be conducted within specific periods of the life span. In developed countries, the ages between 30 and 45 are, for many people, the most demanding, intense, and reinforcing years of adulthood, including events such as having to negotiate the demands of progressing in their chosen career, maintaining an intimate partner relationship, and caring for children [36]. The most important developmental tasks of midlife are generally maintaining professional productivity, mentoring the next generation, parenting adolescent children or emerging adults, and caring for older parents or adjusting to their loss [36,37].

Milestones of adulthood, such as work status, marriage, or parenthood, have traditionally been considered major life events that cue entry into a different life stage [38]. In addition, the structure and demographics of adult life have changed significantly in the past half-century, where the events that have traditionally marked the transition to adulthood, such as completing education, obtaining stable work, parenthood, and marriage, now come later than ever before in many countries [39]. The increase in longevity over the past centuries, especially in Western societies, gives individuals a greater perspective of future time. This, together with globalization granting individuals more perceived choices as well as the weakening of age-related social norms and expectations, offers a wide range of possibilities and time on how to live life and achieve goals during adulthood [37,40]. Although this can be experienced as liberating, it can also be more demanding as individuals have more options to consider but fewer clear guidelines on when and how to pursue their goals [37]. There is evidence that, in the last decade, adults report experiencing more stressors, greater stress severity, greater distress, and more perceived risk for future plans than those two decades ago [38].

In the study of stress and well-being in adulthood, it is important to take gender into account because, as Kennedy et al. state, “Gender powerfully determines all aspects of health and well-being” ([41], p. e1473). According to the World Health Organization (WHO), “Gender refers to the array of socially constructed roles and relationships, personality traits, attitudes, behaviours, values, relative power and influence that society ascribes to the two sexes on a differential basis” [42]. As Heise et al. argue, gender is not exactly apprehended by the long-established male and female dichotomy, instead gender “is a complex social system that structures the life experience of all human beings” ([43], p. 393). It is a social system that characterizes women and men as different and gives resources, roles, power, and entitlements based on that difference, ascribing greater value to men and the conditions considered to be masculine than to women or conditions considered to be feminine [43]. Therefore, the main aim of the present work is to investigate the relevance of gender in the association of stress with eudaimonic well-being and life satisfaction in adulthood. In addition to presenting and comparing the data of men and women on stress, well-being, masculine/instrumental and feminine/expressive traits, coping styles, and social support, the present study conducts an analysis of predictors of eudaimonic well-being and life satisfaction independently in the samples of men and women.

## 2. Materials and Methods

### 2.1. Participants

A cross-sectional study was conducted with 1578 women and 1507 men from the Spanish general population aged between 30 and 59 years. The mean age for men was 44.42 years (*SD* = 8.87) and for women 45.00 (*SD* = 8.24). All participants were volunteers and did not receive financial compensation for their participation. The present study focuses on adulthood, a period that has been generally overlooked [36]. Within adulthood, two periods are generally distinguished: established adulthood, which is estimated to last between 30 and 45 years [36], and midlife, which is estimated to last between 45 and 60 years.

### 2.2. Procedure

This study is part of a larger research project on gender and health in Spain throughout the life cycle from adolescence to old age, and formal approval was obtained from the Ethics Committee on Animal Research and Welfare of the University of La Laguna (study approval number 2015-0170).

Data collection for the larger research project was conducted between 2016 and 2020 by the research team and undergraduate and graduate students of psychology, sociology, and nursing from seven Spanish universities. Students were trained in data collection and received course credits for their participation. To avoid systematic bias, access to participants was through various work and educational centres, both public and private, and also through recreational centres and citizens’ associations located in all the Spanish autonomous communities as well as by resorting to the social network (generally acquaintances, relatives, friends, or neighbours) of the students who collaborated in data collection. All questionnaires and scales were completed individually, manually, and in paper format, without using names or any other data that could identify the participants in the measures. The researcher or collaborating student in charge of administering the tests approached the person who agreed to be part of the research and, after obtaining their informed consent, gave her/him the booklet with the questionnaires, scales, and sociodemographic data collection sheet, explaining how to complete each one of them, noting that it was important for the research that all the items in the questionnaires and scales were answered, and agreed with her/him a date and place for their collection once completed, ensuring that the time interval for completion did not exceed two weeks. The requirements for inclusion in the overall sample were: (1) to be 13 years of age or older and (2) to read, speak, and understand Spanish correctly.

The sample of the present study was randomly selected from the overall sample cited using the following criteria: (1) age between 30 and 59 years and (2) no statistically significant differences in age between men and women.

The study was conducted in accordance with the Declaration of Helsinki and the American Psychological Association Ethical Principles. No names or any other data that would allow identification of the participants were recorded. All participants gave their informed consent before completing the questionnaires and scales and were able to cancel their participation in the study at any time.

### 2.3. Measures

#### 2.3.1. Stress

Following the recommendation to measure several types of stressors [6,7], stress was assessed by means of three instruments: (1) Life Events Questionnaire [44], which is made up of 27 items evaluating the presence or absence during the previous year of changes and/or events in the areas of work, personal, family, partner, financial, legal, illness, geographical moves, death of a loved one, and victimization by violence. Higher scores indicate a greater number of life events during the previous year; (2) Chronic Stress Questionnaire [45], an open-ended instrument in which participants give information about the relative long-lasting problems and threats that they currently face, evaluating the severity of these on a three-point scale ranging from 1 (“not very important”) to 3 (“very important”) where total score is computed by adding the severity response given to each problem and threat considered, with higher scores indicating greater chronic stress; (3) Minor Daily Hassles Questionnaire [45], an open-ended instrument in which the person is asked to write down the daily demands, irritations, and micro-stressors they are currently experiencing, evaluating each on a three-point scale ranging from 1 (“not very important”) to 3 (“very important”). The total score is computed by adding the importance response given to each demand or situation cited, with higher scores indicating the presence of more daily hassles.

#### 2.3.2. Eudaimonic Well-Being

Eudaimonic well-being was assessed using the Spanish version of the Ryff’s Psychological Well-being scale [46]. It is made up of 38 items structured into six factors (self-acceptance, positive relationships, autonomy, environmental mastery, purpose in life, and personal growth) and a second-order latent construct [46]. The response format is a six-point Likert scale ranging from 1 (strongly disagree) to 6 (strongly agree) where higher scores indicate higher eudaimonic well-being. For the current sample, the internal consistency of the 38 items was 0.92.

#### 2.3.3. Life Satisfaction

Life satisfaction was assessed with Life Satisfaction Scale [47]. It is made up of 5 items rated on a seven-point Likert-type scale from 1 (completely disagree) to 7 (completely agree) where higher scores indicate greater level of life satisfaction. This scale has been validated in many countries, including Spain, where it has shown good psychometric properties [48]. For the current sample, the internal consistency of the 5 items was 0.86.

#### 2.3.4. Masculine/Instrumental and Feminine/Expressive Traits

The Bem Sex Role Inventory (BSRI) [49] was used to assess the participants’ masculine/instrumental and feminine/expressive traits. The BSRI is one of the most widely used instruments worldwide to study the extent to which people self-identify with characteristics and traits stereotypically considered appropriate for each gender [50]. This inventory is composed of 60 items consisting of adjectives or short statements, 20 of which refer to socially desirable characteristics stereotypically considered masculine; 20 to socially desirable characteristics stereotypically considered feminine; and the other 20 to characteristics attributable to both genders. Items were scored on a seven-point Likert scale ranging from 1 (never or almost never true) to 7 (always or almost always true) with higher scores indicating greater self-attribution of the trait by the respondent. In the present sample, the internal consistency of the 20 items assessing the masculine/instrumental trait was 0.83 and that of the 20 items assessing the feminine/expressive trait was 0.81.

#### 2.3.5. Coping Styles

Coping styles were assessed using the Spanish version of the Coping Styles Questionnaire (CSQ) [51]. This instrument consists of 47 items that try to capture the typical way of coping with stress. The response format is a four-point Likert scale ranging from never, scored as 0, to always, which is scored as 3. The CSQ is structured into three factors: (1) rational coping, consisting of 15 items whose internal consistency for the current sample was 0.86; (2) emotional coping, consisting of 16 items whose Cronbach’s alpha for the current sample was 0.82; (3) detachment/avoidance coping, consisting of 15 items whose Cronbach’s alpha for the current sample was 0.76.

#### 2.3.6. Social Support

The Social Support Scale [52] was used to measure social support. This scale consists of 12 items that assess perceived social support [53]. Items were scored on a four-point Likert scale ranging from 0 (never) to 3 (always), and higher scores indicate greater levels of social support. The scale is structured in two factors: (1) emotional social support, consisting of 7 items whose internal consistency for the current sample was 0.87; (2) instrumental social support, consisting of 5 items whose internal consistency for the current sample was 0.84.

In addition, a sociodemographic data collection sheet was used to collect information on sociodemographic characteristics, such as age, education, profession, marital status, and number of children.

### 2.4. Statistical Analyses

Statistical analyses were conducted with SPSS 22.0 (IBM Corporation, Armonk, NY, USA) software. Internal consistency was measured using Cronbach’s alpha coefficient. Descriptive analyses were carried out to understand the sociodemographic characteristics of the participants. Comparisons between men and women were computed by using Pearson’s Chi-squared test in case of categorical variables and by using Student´s *t*-test when the variables were continuous. Effect size of the mean differences in the study variables was computed using Cohen’s *d* [54]. The bivariate associations between the study variables were calculated using Pearson’s *r* correlation coefficient, except for the educational level where Spearman’s Rho was used, as it is an ordinal variable. Five-step hierarchical multiple regression analyses were used to determine the relevance of the stress, the masculine/instrumental and feminine/expressive traits, the coping styles, the self-esteem, and the social support in men’s and women’s eudaimonic well-being and life satisfaction. In each regression analysis, the respondents’ age, number of children, and education, as an ordinal variable with 8 levels (from 0 for basic education to 7 for five-year university degree) were entered in Model 1 to control their effect; scores on chronic stress, minor daily hassles, and number of life events experienced in the last year were added in Model 2; masculine/instrumental and feminine/expressive traits scores were added in Model 3; coping styles scores were added in Model 4; finally, the scores on emotional and instrumental social support were added in Model 5. For all analyses, *p*-values less than 0.05 were considered statistically significant.

## 3. Results

### 3.1. Sociodemographic Characteristics

Table 1 displays the main sociodemographic characteristics and the comparison for the men and women. As can be seen, there were statistically significant differences in all variables except for age. Although there was diversity in educational level, 32.6% of the men and 37.8% of the women had a university education. There was also diversity in occupation, with men being more likely than women to have manual or non-manual occupations, and there were only “housewife” women, which occurred in 11.9% of the women. The most frequent marital status was married or cohabiting, which was found in 68.9% of the men and 69% of the women. The number of children ranged from none, which was found in 30.1% of men and 20.7% of women, to eight, which was found in 0.1% of men and women, with the most frequent being two children (37.4% of men and 42.8% of women) or one child (21.5% of men and 22.4% of women).

### 3.2. Comparisons for Men and Women for Well-Being Measures, Stress, Masculine/Instrumental and Feminine/Expressive Traits, Coping Styles, and Social Support

Table 2 displays the means, standard deviations, and men and women comparisons for the well-being measures, stress, masculine/instrumental and feminine/ expressive traits, coping styles, and social support. Statistically significant differences were found in seven of twelve variables, although effect sizes were small except for masculine/instrumental and feminine/expressive traits where effect size was medium. Women scored higher than men in chronic stress, minor daily hassles, feminine/expressive trait, emotional coping style, and instrumental social support. Men scored higher than women in masculine/instrumental trait and rational coping style.

### 3.3. Correlations between Study Variables and Eudaimonic Well-Being and Life Satisfaction

For both men and women, the three stress measures were positively and statistically significantly correlated (*p* < 0.001). In men, the number of life events during the last year correlated 0.20 with chronic stress and 0.19 with minor daily hassles, resulting in a correlation coefficient between chronic stress and minor daily hassles of 0.53. In women, the correlation coefficients were 0.25, 0.18, and 0.52, respectively. The three coping styles were also statistically significantly correlated (*p* < 0.001) although the effect size was small or medium. In men, their emotional coping style correlated −0.20 with the rational coping style and 0.30 with the detachment/avoidance coping style while rational coping style correlated 0.24 with detachment/avoidance coping style. In women, the correlation coefficients were −0.29, 0.10, and 0.34, respectively. Eudaimonic well-being was positively correlated with life satisfaction; the correlation coefficient for men was 0.56 (*p* < 0.001), and for women, it was 0.60 (*p* < 0.001).

Table 3 presents bivariate correlations of the study variables with eudaimonic well-being and life satisfaction for men and women. As can be seen, in both genders, most of the correlation coefficients were statistically significant although the magnitude of the association of eudaimonic well-being and life satisfaction with the sociodemographic variables and with most of the stress measures as well as with detachment/avoidance coping style were small.

### 3.4. Hierarchical Multiple Linear Regression Analyses Predicting Eudaimonic Well-being and Life Satisfaction

Table 4 provides the main results of the hierarchical multiple linear regression analyses with eudaimonic well-being as the dependent variable for men; Table 5 provides these for women. Results identified that in both men and women, *R* for regression was significantly different from zero at the end of the five models. After Model 1, introducing age, the number of children, and educational level in the equation, *R*^2^ = 0.02 in men and *R*^2^ = 0.04 in women. The addition of the three stress measures to the equation (Model 2) resulted in an *R*^2^ change = 0.02 in men and an *R*^2^ change = 0.03 in women. Beta values were only statistically significant for chronic stress (β = −0.13, *p* < 0.001 for men and β = −0.08, *p* < 0.01 for women) and the number of life events during the last year (β = −0.09, *p* < 0.01 for men and β = −0.14, *p* < 0.001 for women). The change in *R*^2^ from Model 2 to Model 3 identified that the masculine/instrumental trait (β = 0.32, *p* < 0.001) and the feminine/expressive trait (β = 0.14, *p* < 0.001) for men, and masculine/instrumental trait (β = 0.33, *p* < 0.001) and feminine/expressive trait (β = 0.15, *p* < 0.001) for women play a significant role in eudaimonic well-being. The addition of coping styles in Model 4 resulted in an important increment in *R*^2^ (*R*^2^ change = 0.32 in men and *R*^2^ = 0.35 in women). Beta values were only statistically significant for the emotional coping style (β = −0.46, *p* < 0.001 for men and β = −0.44, *p* < 0.001 for women) and the rational coping style (β = 0.30, *p* < 0.001 for men and β = 0.34, *p* < 0.001 for women). The addition of social support measures to the equation (Model 5) resulted in an *R*^2^ change = 0.06 in men and an *R*^2^ change = 0.05 in women. Beta values in Model 5 with all independent variables in the equation proved that a low emotional coping style and high rational coping style were the variables most associated with men’s and women’s higher eudaimonic well-being. In men, the third most relevant variable was high masculine/instrumental trait followed by high emotional social support, high instrumental social support, high feminine/expressive trait, and low chronic stress. In women, the third most relevant variables were high scores in masculine/instrumental trait and emotional social support followed by high instrumental social support, a high educational level, and high feminine/expressive trait. The final model accounted for a total of 56.6% of the variance in eudaimonic well-being in men and 61.2% in women.

Table 6 presents the main results of the hierarchical multiple linear regression analyses with life satisfaction as the dependent variable for men whereas Table 7 corresponds to women. Results identified that, in both genders, *R* for regression was significantly different from zero at the end of the five models. After Model 1, with age, the number of children, and educational level in the equation, *R*^2^ = 0.02 in men and *R*^2^ = 0.03 in women. The addition of the stress measures to the equation (Model 2) resulted in an *R*^2^ change = 0.04 in men and an *R*^2^ change = 0.08 in women. The change in *R*^2^ from Model 2 to Model 3 identified that masculine/instrumental and feminine/expressive traits resulted in an *R*^2^ change = 0.08 in men and *R*^2^ = 0.05 in women. The addition of the three coping styles in Model 4 also resulted in an increment in *R*^2^ (*R*^2^ change = 0.10 in men and *R*^2^ = 0.14 in women). Beta values were statistically significant (*p* < 0.001) for the three coping styles: β = −0.27 for men and β = −0.32 for women for the emotional coping style; β = 0.14 for men and β = 0.12 for women for the rational coping style; lastly, β = 0.10 for men and also β = 0.10 for women for the detachment/avoidance coping style. The addition of social support measures to the equation (Model 5) resulted in an *R*^2^ change = 0.05 in both groups. Beta values in Model 5 with all independent variables in the equation proved that a low emotional coping style and high emotional social support were the variables most associated with men’s and women’s higher life satisfaction. In men, the third most relevant variable was high instrumental social support followed by high scores in masculine/instrumental and in feminine/expressive traits, high rational and detachment/avoidance coping styles, more children, low chronic stress, a smaller number of life events during the last year, and a high educational level. In women, the third most relevant variable was a smaller number of life events during the last year followed by high instrumental social support, a high detachment/avoidance coping style, more children, a high educational level, a high rational coping style, high scores in masculine/instrumental and in feminine/expressive traits, fewer minor daily hassles, and a younger age. The final model accounted for a total of 28% of the variance in life satisfaction in men and 34.3% in women.

## 4. Discussion

The main aim of this study was to investigate the relevance of gender in the association of stress with eudaimonic well-being and life satisfaction in adulthood. We also compared men’s and women’s scores on stress, well-being, masculine/instrumental and feminine/expressive traits, coping styles, and social support and analysed predictors of eudaimonic well-being and life satisfaction independently in men and women. Stress was assessed by three measures: chronic stress, minor daily hassles, and the number of life events during the last year. Regression analyses showed that in both women and men, higher chronic stress and a higher number of life events during the last year predicted lower eudaimonic well-being. However, when coping styles were included in the equation, none of the stress measures was significantly associated with women’s eudaimonic well-being, suggesting that such coping styles buffer the negative effect that stress has on women’s eudaimonic well-being. In men, the inclusion of coping styles in the equation made the number of life events no longer a statistically significant predictor of eudaimonic well-being, and the influence of chronic stress was reduced. The effect of chronic stress was maintained when social support was introduced into the equation, and, in the final model with all variables in the equation greater chronic stress was a statistically significant predictor of men’s lower eudaimonic well-being although it contributed modestly to that prediction.

When predicting men’s life satisfaction, it was found that higher chronic stress and a higher number of events during the last year were associated with lower life satisfaction, and the two stress measures remained statistically significant when coping styles and social support were included in the equation. In the women’s sample, all three stress measures were statistically significant predictors of life satisfaction, although the number of life events experienced during the last year was more influential. When coping styles were included in the equation, chronic stress was no longer statistically significant. In the final model with all variables in the equation the main predictor of women’s higher life satisfaction was a lower emotional coping style followed by higher emotional support and a lower number of events during the previous year. Minor daily hassles were also associated with life satisfaction, but their predictive power was lower. In summation, stress does not seem to affect women’s sense of meaning and purpose in life, but a greater number of life events and more minor daily hassles are associated with lower women’s life satisfaction. On the contrary, in men, greater chronic stress is the form of stress that most jeopardizes their well-being, being associated with a lower sense of meaning and purpose in life and with lower life satisfaction; in addition, a greater number of life events is also associated with men’s lower life satisfaction, although its influence on life satisfaction is less than in women. Although the reason for these differences is unknown, it could be due to the fact that women, following gender norms and roles, are more involved than men in family roles and household chores, which may be an important source of daily hassles. Such greater involvement, in addition to generating more daily hassles for them than for men, could cause them to attribute greater relevance to daily hassles. Although daily hassles have been considered to be relatively transient and seemingly minor, it has been suggested that they may exert both long- and short-term effects on individuals’ well-being [37].

The analysis of the relevance of the internalization of traits and characteristics stereotypically associated with masculinity and femininity on women’s and men’s well-being showed that their relevance differed according to the type of well-being considered. When predicting eudaimonic well-being, although both traits were statistically significant in both women and men, the masculine/instrumental trait was much more relevant. Specifically, in the final model, the beta weight was 0.20 in men and 0.19 in women for the masculine/instrumental trait and 0.07 in men and 0.06 in women for the feminine/expressive trait. When predicting life satisfaction, in the final model, the significance of both traits was similar but higher for men (β = 0.12 for masculine/instrumental and β = 0.11 for feminine/expressive) than for women (β = 0.07 for masculine/instrumental and β = 0.06 for feminine/expressive). These results do not support gender norms and stereotypes that consider women and men to be essentially different [43,55], and it is the masculine male and the feminine female who typify psychological health [56].

The results of the differential analyses showed that there were statistically significant gender differences in only seven of the twelve variables analysed, and when statistically significant differences were found, the effect size was small or trivial except for masculine/instrumental and feminine/expressive traits where effect sizes were medium. These results cohere with those of the meta-synthesis by Zell et al. [57] where, after evaluating psychological gender differences through the results of 106 meta-analyses including data from 12,238,667 participants, they found that most effects sizes were small (between 0.11 and 0.35) or very small (between 0 and 0.10). The largest differences were found in masculine versus feminine traits where the effect size was medium. These results add to the evidence that, against the prevailing lay assumption and gender stereotypes that men and women are greatly different, there are few gender differences, with the magnitude of differences being generally small in adulthood. One exception is the greater self-identification of men as opposed to women with characteristics and traits traditionally considered masculine and of women as opposed to men with characteristics and traits traditionally considered feminine. This can be interpreted as, despite the social changes and advances towards gender equality that have taken place in Western societies, gender norms and stereotypes still continue to have some relevance in women’s and men’s self-concept.

Our results on gender differences in stress showed that, although there were no statistically significant gender differences in life events, women showed more chronic stress and daily hassles than men, results that agree with those of other studies [19,58,59] and with those of studies conducted in other stages of the life cycle, such as emerging adulthood [20] and old age [60]. In addition, as shown in previous research [19,20,58,59], it was found that men scored higher than women in the rational coping style and women higher than men in the emotional coping style. Coping styles were very important for women’s and men’s well-being, with more emotional and less rational coping being the most influential predictors of both women’s and men’s lower sense of meaning and purpose in life. Despite a more emotional coping style being the most influential predictor of both women’s and men’s lower life satisfaction, social support was more influential in life satisfaction than a rational coping style. In both women and men, a greater detachment/avoidance coping style was as influential as a greater rational coping style in predicting greater life satisfaction. The relevance of coping for achieving well-being has been found in other works where emotion-focused coping has been identified as the one with a negative and highest predictive power of well-being [60].

This study had some limitations. First, our study used a cross-sectional design so cause–effect relationships cannot be established. Second, the sample, although large and with participants with different sociodemographic characteristics, is not a random sample. The sampling method used does not seem to guarantee that the research sample is representative of the Spanish general population, preventing the generalizability of the results. Third, all the variables were assessed by means of self-report measures, which could introduce bias in the information received, such as social desirability and/or recall bias. Finally, although different types of well-being were assessed, affective well-being was not included.

Despite these limitations, the current study has important strengths, including the use of various measures of stress and well-being; the study focus on the analysis of a specific period of the life cycle; and the large sample size that includes people of different sociodemographic characteristics and a fairly similar percentage of women and men. Furthermore, it is research that has been conducted following a gender perspective, which has made it possible to add to the literature on the relevance of gender norms and roles in the stress and well-being of adult women and men.

The present study’s results allow us to conclude that gender is relevant in the stress and well-being of adults as well as in the association between stress and well-being. Results showed that in women’s and men’s s well-being, stress coping styles are very relevant and that while a high rational coping style is associated with greater well-being, conversely, a high emotional style is associated with less well-being. These results are relevant for the design of programs and strategies aimed at better stress management and promoting women’s and men’s well-being, encouraging a more rational and less emotional coping style. In contrast to gender norms and stereotypes that consider women and men to be different and complementary, with men being and having to be masculine/instrumental but not feminine/expressive and women being and having to be feminine/expressive but not masculine/instrumental, the results of the present study have shown that both traits, masculine/instrumental and feminine/expressive, are associated with women’s and men’s well-being. These results are relevant for the design of policies and programs to increase gender equality.

## Figures and Tables

**Table 1:** Demographic characteristics of men and women.

	Men (n = 1507)		Women (n = 1578)		χ^2^ -Value
	n	%	n	%	
*Educational level*					
Unfinished elementary studies	15	1.0	16	1.0	17.14 **
Elementary studies	353	23.4	375	23.8	
First-grade professional training	122	8.1	143	9.1	
High school degree	327	21.7	277	17.6	
Second-grade professional training	199	13.2	171	10.8	
University degree	491	32.6	596	37.8	
*Occupation*					
Skilled/unskilled manual	542	38.2	442	29.1	215.90 ***
Skilled non-manual	510	35.9	433	28.5	
Professional	334	23.5	445	29.3	
Self-employment	34	2.4	17	1.1	
Housewife	0	0.0	181	11.9	
Non-data	147		60		
*Marital status*					
Never married	354	23.6	263	16.7	55.69 ***
Married/cohabiting	1034	68.9	1088	69.0	
Separated/divorced	107	7.1	199	12.6	
Widowed	5	0.3	27	1.7	
Non-data	7		1		
	***M***	***SD***	***M***	***SD***	***t*-Value**
Age	44.42	8.87	45.00	8.24	−1.89
Number of children	1.32	1.08	1.53	1.05	−5.61 ***

*Note:* ** *p* < 0.01; *** *p* < 0.001.

**Table 2:** Mean (*M*), standard deviation (*SD*), and comparisons for men and women groups for well-being, stress, masculine/instrumental and feminine/expressive traits, coping styles, and social support.

Variable	Men (n = 1507)		Women (n = 1578)		*t*-Value	*d*-Value
	*M*	*SD*	*M*	*SD*		
Eudaimonic well-being	172.16	22.96	171.69	24.11	0.55	0.02
Life satisfaction	24.31	5.98	24.04	6.42	1.19	0.04
Chronic stress	4.55	3.90	4.94	4.28	−2.65 **	−0.11
Minor daily hassles	3.87	3.65	4.92	4.14	−7.46 ***	−0.27
Number of life events during the last year	2.56	2.24	2.55	2.22	0.77	0.00
Masculine/instrumental trait	96.05	14.29	87.05	14.63	17.27 ***	0.62
Feminine/expressive trait	95.82	12.26	102.05	12.01	−14.26 ***	−0.51
Emotional coping style	13.33	5.89	15.00	6.37	−7.55 ***	−0.27
Rational coping style	27.25	7.06	25.65	6.66	6.45 ***	0.21
Detachment/avoidance coping style	16.61	5.80	16.22	5.66	−1.89	-0.07
Emotional support	17.00	4.07	16.96	4.16	0.32	0.01
Instrumental support	9.77	3.87	10.58	3.65	−6.00 ***	−0.22

*Note:* ** *p* < 0.01; *** *p* < 0.001.

**Table 3:** Correlations of study variables with eudaimonic well-being and life satisfaction for men and women.

	Men		Women	
	*Eudaimonic Well-Being*	*Life Satisfaction*	*Eudaimonic Well-Being*	*Life Satisfaction*
Age	−0.10 ***	−0.02	−0.06 *	−0.04
Educational level ^$^	0.12 ***	0.10 ***	0.20 ***	0.16 ***
Number of children	−0.03	0.06 *	−0.04	0.04
Chronic stress	−0.11 ***	−0.15 ***	−0.11 ***	−0.18 ***
Minor daily hassles	−0.04	−0.07 **	−0.06 *	−0.15 **
Number of life events during the last year	−0.09 **	−0.14 ***	−0.16 **	−0.26 ***
Masculine/instrumental trait	0.37 ***	0.24 ***	0.37 ***	0.19 ***
Feminine/expressive trait	0.24 ***	0.23 ***	0.21 ***	0.15 ***
Emotional coping style	−0.54 ***	−0.30 ***	−0.59 ***	−0.43 ***
Rational coping style	0.49 ***	0.30 ***	0.55 ***	0.30 ***
Detachment/avoidance coping style	−0.02	0.09 **	0.09 **	0.12 **
Emotional social support	0.48 ***	0.37 ***	0.51 ***	0.42 ***
Instrumental social support	0.43 ***	0.35 ***	0.46 ***	0.40 ***

*Notes:*^$^ Coefficients calculated with Spearman Rho; * *p* < 0.05; ** *p* < 0.01; *** *p* < 0.001.

**Table 4:** Summary of the hierarchical regression with eudaimonic well-being as the dependent variable for men.

	Model 1		Model 2		Model 3		Model 4		Model 5	
	β	*t*-Value	β	*t*-Value	β	*t*-Value	β	*t*-Value	β	*t*-Value
Age	−0.13	−4.18 ***	−0.15	−4.93 ***	−0.08	−3.02 **	−0.07	−3.10 **	−0.03	−1.27
Number of children	0.05	1.65	0.05	1.72	0.02	0.72	0.02	1.11	0.02	0.98
Educational level	0.11	4.37 ***	0.11	4.20 ***	0.10	4.32 ***	0.03	1.53	0.02	1.21
Chronic stress			−0.13	−4.18 ***	−0.10	−3.46 **	−0.05	−2.46 **	−0.05	−2.20 **
Minor daily hassles			0.04	1.41	0.02	0.80	0.01	0.37	0.02	0.84
Number of life events during the last year			−0.09	−3.23 **	−0.10	−4.22 **	−0.02	−0.87	−0.00	−0.11
Masculine/instrumental trait					0.32	12.71 ***	0.20	9.64 ***	0.20	10.24 ***
Feminine/expressive trait					0.14	5.66 ***	0.12	5.95 ***	0.07	3.81 ***
Emotional coping style							−0.46	−22.23 ***	-0.39	−19.98 ***
Rational coping style							0.30	14.28 ***	0.26	12.97 ***
Detachment/avoidance coping style							−0.00	−0.02	-0.00	−0.16
Emotional social support									0.18	7.49 ***
Instrumental social support									0.11	4.43 ***
*R^2^*	0.02		0.05		0.19		0.51		0.57	
*R^2^* Change	0.02 ***		0.02 ***		0.14 ***		0.32 ***		0.06 ***	

*Note:* β = Standardized regression coefficient. *R^2^* = percentage of explained variance. ** *p* < 0.01; *** *p* < 0.001.

**Table 5:** Summary of the hierarchical regression with eudaimonic well-being as the dependent variable for women.

	Model 1		Model 2		Model 3		Model 4		Model 5	
	β	*t*-Value	β	*t*-Value	β	*t*-Value	β	*t*-Value	β	*t*-Value
Age	−0.06	−2.03 *	−0.08	−2.93 **	−0.08	−2.97 **	−0.07	−3.49 ***	−0.02	−1.00
Number of children	0.02	0.76	0.02	0.77	0.03	1.16	0.02	1.03	0.02	1.03
Educational level	0.19	7.74 ***	0.18	7.30 ***	0.18	7.86 ***	0.10	6.05 ***	0.09	5.28 ***
Chronic stress			−0.08	−2.76 **	−0.06	−2.32 *	−0.01	−0.44	−0.01	−0.40
Minor daily hassles			0.01	0.16	0.01	0.28	−0.01	−0.25	0.00	0.20
Number of life events during the last year			−0.14	−5.25 ***	−0.15	−6.26 ***	−0.04	−2.42 *	−0.01	−0.76
Masculine/instrumental trait					0.33	14.45 ***	0.19	10.53 ***	0.19	11.20 ***
Feminine/expressive trait					0.15	6.48 ***	0.10	6.05 ***	0.06	3.53 ***
Emotional coping style							−0.44	−23.40 ***	−0.36	−19.85 ***
Rational coping style							0.34	17.49 ***	0.31	16.56 ***
Detachment/avoidance coping style							−0.01	−0.75	−0.01	−0.70
Emotional social support									0.19	7.33 ***
Instrumental social support									0.09	3.76 ***
*R^2^*	0.04		0.07		0.22		0.57		0.62	
*R^2^* Change	0.04 ***		0.03 ***		0.15 ***		0.35 ***		0.05 ***	

*Note:* β = Standardized regression coefficient. *R^2^* = percentage of explained variance. * *p* < 0.05; ** *p* < 0.01; *** *p* < 0.001.

**Table 6:** Summary of the hierarchical regression with life satisfaction as the dependent variable for men.

	Model 1		Model 2		Model 3		Model 4		Model 5	
	β	*t*-Value	β	*t*-Value	β	*t*-Value	β	*t*-Value	β	*t*-Value
Age	−0.08	−2.62 **	−0.11	−3.64 ***	−0.07	−2.29 *	−0.06	−2.16 *	−0.02	−0.75
Number of children	0.11	3.64 **	0.11	3.72 ***	0.09	3.17 **	0.10	3.60 ***	0.09	3.60 ***
Educational level	0.11	4.46 ***	0.11	4.23 ***	0.10	4.31 ***	0.07	3.06 **	0.06	2.83 **
Chronic stress			−0.15	−4.86 ***	−0.13	−4.40 ***	−0.10	−3.59 ***	−0.09	−3.40 **
Minor daily hassles			0.03	0.94	0.02	0.62	0.01	0.47	0.02	0.81
Number of life events during the last year			−0.12	−4.49 ***	−0.13	−5.13 ***	−0.08	−3.50 ***	−0.07	−2.99 **
Masculine/instrumental trait					0.18	7.11 ***	0.12	4.55 ***	0.12	4.68 ***
Feminine/expressive trait					0.17	6.76 ***	0.15	6.16 ***	0.11	4.48 ***
Emotional coping style							−0.27	−10.55 ***	−0.21	−8.36 ***
Rational coping style							0.14	5.17 ***	0.10	3.81 ***
Detachment/avoidance coping style							0.10	3.94 ***	0.10	3.99 ***
Emotional social support									0.16	5.03 ***
Instrumental social support									0.13	3.53 ***
*R^2^*	0.02		0.06		0.14		0.23		0.29	
*R^2^* Change	0.02 ***		0.04 ***		0.08 ***		0.10 ***		0.05 ***	

*Note:* β = Standardized regression coefficient. *R^2^* = percentage of explained variance. * *p* < 0.05; ** *p* < 0.01; *** *p* < 0.001.

**Table 7:** Summary of the hierarchical regression with life satisfaction as the dependent variable for women.

	Model 1		Model 2		Model 3		Model 4		Model 5	
	β	*t*-Value	β	*t*-Value	β	*t*-Value	β	*t*-Value	β	*t*-Value
Age	−0.08	−2.76 **	−0.12	−4.33 ***	−0.11	−4.24 ***	−0.11	−4.39 ***	−0.06	−2.51 *
Number of children	0.10	3.55 **	0.10	3.71 ***	0.10	3.87 ***	0.10	4.22 ***	0.10	4.33 ***
Educational level	0.17	6.74 ***	0.15	6.17 ***	0.15	6.46 ***	0.11	5.14 ***	0.09	4.36 ***
Chronic stress			−0.09	−3.26 **	−0.08	−2.98 **	−0.04	−1.53	−0.04	−1.53
Minor daily hassles			−0.06	−2.24 *	−0.06	−2.27 *	−0.07	−2.64 **	−0.06	2.41 *
Number of life events during the last year			−0.22	−8.98 ***	−0.23	−9.53 ***	−0.16	−7.10 ***	−0.13	−5.97 ***
Masculine/instrumental trait					0.15	6.50 ***	0.08	3.29 **	0.07	3.39 **
Feminine/expressive trait					0.13	5.38 ***	0.10	4.47 ***	0.06	2.53 *
Emotional coping style							−0.32	−13.54 ***	−0.25	−10.48 ***
Rational coping style							0.12	4.81 ***	0.09	3.57 ***
Detachment/avoidance coping style							0.10	4.45 ***	0.10	4.62 ***
Emotional social support									0.15	4.54 ***
Instrumental social support									0.12	3.68 ***
*R^2^*	0.03		0.12		0.16		0.30		0.35	
*R^2^* Change	0.03 ***		0.08 ***		0.05 ***		0.14 ***		0.05 ***	

*Note:* β = Standardized regression coefficient. *R^2^* = percentage of explained variance. * *p* < 0.05; ** *p* < 0.01; *** *p* < 0.001.

## Data Availability

The datasets used and analysed within the framework of this study are available from the corresponding author on reasonable request.
